# STR typing of skin swabs from individuals after an allogeneic hematopoietic stem cell transplantation

**DOI:** 10.1007/s00414-022-02847-5

**Published:** 2022-06-03

**Authors:** Dagmar von Máriássy, Roland Reibke, Mareike Verbeek, Britta Gätjens, Roberta Schiller, Katja Anslinger

**Affiliations:** 1 Institute of Legal Medicine, Ludwig-Maximilians-University, Nußbaumstr. 26, 80336 Munich, Germany; 2 Department of Internal Medicine I, Klinikum Bad Trissl, Oberaudorf, Germany; 3 Department of Internal Medicine III, Klinikum Rechts Der Isar, Technische Universität München, Munich, Germany

**Keywords:** Molecular genetic investigation, STR typing, Allogeneic hematopoietic stem cell transplantation (alloHSCT), Langerhans cells (LC), Graft versus host disease (GvHD)

## Abstract

One of the pre-requisites for forensic DNA analysis is the fact that all nucleated cells of a person carry the same genetic information. However, this is not the case for individuals who have received an allogeneic hematopoietic stem cell or bone marrow transplantation, as all new cells formed by the bone marrow no longer show the genetic information of the recipient but that of the donor, while all other cells still carry the original information before transplantation. Thus, STR typing of a blood sample after successful transplantation yields a DNA profile that differs from the recipient’s original profile and corresponds to the donor genotype instead. Evidence from a routine case suggests that transplanted individuals may show donor alleles in skin swabs, as well. In order to examine this issue more closely, various skin swabs from 28 patients who have received an allogeneic hematopoietic stem cell transplantation were examined in this study. Swabs from the right and left palm, the back of the hand, one of the two upper arms, and the neck were collected from each person. Ninety-one of the 140 resulting swabs delivered useful results. All of those samples showed mixtures of recipient and donor DNA with different mixture ratios and the proportions of donor and recipient alleles revealed inter- and intra-individual differences. Those results were discussed with respect to graft versus host disease.

## Introduction

The most important basis for a molecular genetic investigation is that the genetic information in every nucleated cell of a person’s body is identical. If a person leaves cell material such as skin cells and blood or mucosal cells behind on objects or at location, STR typing yields identical DNA profiles that can usually be assigned to that person.

People, who in addition to their own hereditary traits carry the genetic information of another person, are an exception. Whereas a so-called chimerism can occur after every allogeneic organ transplantation, it is rarely found after transplantation of solid organs. A bone marrow or allogeneic hematopoietic stem cell transplantation (alloHSCT) aims to completely substitute the recipient’s endogenous bone marrow with that of the donor. Accordingly, all blood and immune cells built from the new bone marrow show the donor’s characteristics (complete chimerism). Allogeneic hematopoietic stem cell transplantation has become increasingly important in recent years due to a significantly expanded indication for both malignant and none-malignant bone marrow diseases (especially leukemia). The European Society for Blood and Marrow Transplantation (EBMT) registered 19.630 patients with a first-time alloHSCT within their area of competence in 2018 [1]. The number of first transplantations in Germany increases by approximately 3.200 per year, adding to more than 45.000 conducted transplantations since 1998 [2]. Whereas a successfully transplanted recipient’s blood sample cannot be distinguished from that of the donor, non-hematopoietic cells should still exhibit the recipient’s characteristics. The portion of blood or immune cells migrating to solid organs is largely unknown. However, after a successful alloHSCT, the DNA in a patient’s blood sample is usually different from the DNA in the same patient’s other cellular material.

A swab from a person’s hand who had previously received bone marrow transplantation was examined as part of a molecular genetic trace investigation in a sexual offense case. STR typing revealed a DNA mixture of recipient and donor DNA alleles [3]. At this point, the question arose whether those results represent an exception or whether donor alleles can be detected in body swab samples from all persons with a previous alloHSCT or bone marrow transplantation, which would inevitably, affects the interpretation of DNA results in case scenarios involving transplanted persons.

In order to clarify the issue, swabs from different skin areas were collected from 28 transplanted patients in this study. All swabs were pre-tested for the presence of blood. Subsequently, STR profiles were created and, as far as possible, used to determine the proportion of recipient to donor alleles.

## Materials and methods

### Sampling

Cotton swabs (Sarstedt Forensic Swab in transport tubes with ventilation membranes), moistened with H_2_O, were used to collect swabs from the following skin areas of 28 individuals who had received an alloHSCT at least 6 months in advance: the right and left palm, the back of either the right or left hand, the right or left upper arm, and the neck. Swabs were then left to air-dry. Additionally, a buccal swab and a blood sample (aliquots from blood samples taken during outpatient examination) were taken from each person and left to air-dry in order to determine recipient and donor alleles.

Prior to sampling, each person was informed about both scope and aim of the study and gave their written consent. Samples were completely anonymized immediately after sampling and could therefore not be assigned to the respective patient subsequently. Due to the principle of data avoidance and data economy, subsequent patient survey was limited. Neither data regarding a patient’s medical history with special regard to the donor (family/unrelated donor), intensity of prior therapy or pre-transplantation conditioning treatment, and the question of skin reactions as an indication of an immune clearance or graft versus host disease nor data regarding a patient’s personal environment and habits (family status, contact with other persons, handedness, last hand washing, etc.) were collected. Hair was not collected either. Samples were collected at Klinikum Rechts der Isar, Technische Universität München, and München Klinik Schwabing usually by the same doctor under identical conditions. Sampling and sample analysis were performed by different individuals.

### Preliminary blood test

All body swabs and oral swabs were tested for blood in advance using the Combur^3^Test®E (Roche Diagnostics, Mannheim, DE).

### STR typing

After external lysis, DNA was isolated semi-automatically with the Maxwell®16 robotic system and the Maxwell® Blood DNA Kit (Promega, WI, USA), according to the manufacturer’s instructions. The amount of human DNA per sample was determined using the Quantifiler® Trio DNA Quantification Kit (Thermo Fisher Scientific) and a real-time PCR instrument 7500 (Applied Biosystems, Foster City, CA), according to the manufacturer’s instructions. Samples with DNA amounts of less than 0.5 pg/µl were not submitted for subsequent analysis. PCR was conducted for samples with DNA amounts of ≥ 0.5 pg/µl using the PowerPlex® ESX 17 Fast System (Promega, WI, USA) on a 2720 Thermal Cycler (Applied Biosystems, Foster City, CA) in a volume of 12.5 µl (in-house validation). A maximum DNA amount of 300 pg was set per run. Samples ranging from 100 to 300 pg of DNA went through 30 PCR cycles. All samples that contained less than 100 pg of DNA went through 32 PCR cycles. Single DNA fragment analysis was carried out per sample on a 3500xL Genetic Analyzer (Applied Biosystems, Foster City, CA) with the GeneMapper® ID-X Software v1.4 (Applied Biosystems, Foster City, CA) according to the manufacturer’s instructions. Positive and negative controls were included at each step.

### Evaluation

Since cells newly produced by the bone marrow after successful transplantation should exclusively show donor alleles, each patient’s blood sample was used as a reference for the corresponding donor’s DNA profile. Buccal swabs were used as the recipient’s reference sample because usually a high proportion of buccal mucosa cells can be expected in those samples.

For evaluation, results were divided into three categories: Samples that yielded either no or no useful STR typing results, specifically profiles with less than 15 fully typed systems and non-interpretable DNA mixtures, were assigned to category A. All samples from patients whose recipient and donor alleles could be completely or partially derived and which yielded results in 15 or 16 autosomal systems, respectively, were grouped in category B. Finally, category C included all samples that were suitable for calculating the recipient and donor proportion. These were samples from category B in which all recipient and/or donor DNA alleles were present that either did not show any additional alleles from other individuals or, if alleles from other individuals were present, these occurred only sporadically or their intensity was so low that they could be distinguished from the recipient and donor alleles.

#### Calculating the proportion of recipient and donor alleles

The proportion of recipient and donor alleles was calculated based on the peak area ratios across all systems.

#### Statistical calculations

Statistical calculations were performed using IBM® SPSS® Statistics Version 25 software.

## Results and discussion

### Preliminary blood test

Pre-testing for blood was positive or weakly positive for 11 out of 28 buccal swabs. For all remaining buccal swabs and all skin swabs, the test was negative.

### Determination of donor and recipient alleles

#### Blood samples

Since cells newly produced by the bone marrow after successful transplantation should exclusively show the donor’s alleles, blood samples were taken from the patients in order to determine them. For 18 tested persons, STR typing of such blood samples revealed single person profiles that differed from the respective buccal swab results (complete chimerism). Eight blood samples showed mixtures (mixed chimerism), which indicate a possible recurrence. In seven samples, isolated recipient alleles were detected, whereas one sample showed the complete recipient’s DNA profile as a minor component (assignment of donor alleles according to the respective buccal swabs). Two blood samples resulted in balanced two-person-mixtures, whereby the donor characteristics could be derived for one of the samples based on the alleles obtained for the corresponding buccal swab. The donor alleles could not be derived for the other sample (patient 15).

#### Buccal swabs

Only two buccal swabs (reference sample for the recipient’s DNA profile) yielded individual profiles that did not match the blood samples’ alleles, including one sample with a weakly positive preliminary blood test. However, this person showed a complete chimerism in the blood sample. Thus, the presence of the patient’s blood on the according buccal swab, e.g., due to bleeding gums, would have resulted in a mixture of recipient and donor alleles. Consequently, the Combur test result may have been falsely positive, possibly triggered by other substances on the swab, such as food residues. All other buccal swabs showed mixtures in STR typing. For 16 of these samples, however, clearly definable main components could be derived, which in turn did not correspond to the respective alleles of the blood samples. Analysis of the buccal swabs of the remaining ten persons examined resulted in more or less balanced allele mixtures, whereby partial recipient DNA profiles could be derived regarding the donor alleles obtained from the corresponding blood samples. For one individual (patient 15), the recipient alleles could not be deduced due to the mixed genotype in the corresponding blood sample. In all ten buccal swabs that tested positive for blood and showed mixtures, the admixture of donor alleles can be explained by the additional blood cells. For the remaining 16 swabs with visible allele mixtures, the preliminary blood test was negative. This means that either the test showed false negative results or that cells with donor alleles were found on the swabs lacking the admixture of erythrocytes (for further discussion, see the “Determination of ratio of recipient’s to donor’s alleles” section).

Since neither the blood sample nor the buccal swab from patient 15 revealed individual DNA profiles or main components, neither donor nor recipient characteristics could be determined for this individual. Consequently, the skin swabs of this person could not be evaluated with regard to the proportion of recipient or donor alleles and were assigned to category A. Only the quantification results of these samples were included in the evaluation below. All other results exclusively refer to the remaining 27 patients.

### Evaluation of the skin swabs

#### Quantification of DNA

The quantification of human DNA resulted in less than 0.5 pg/µl for 11 swabs (8%). These samples were not further examined. Fourteen percent of the samples contained DNA amounts of 0.5–1 pg/μl, 53% amounts of 1–10 pg/µl, and 22% contained a DNA amount of 10–100 pg/µl. Four samples (3%) contained an amount greater than 100 pg/µl DNA (see Table 1). The highest yield of DNA was obtained from a swab from one person’s neck with 570 pg/µl. The calculated median values of DNA amounts across all collection sites per subject resulted in a range from 0.2–82.1 pg/µl (see Table 1), showing a very large variability in the amount of DNA obtained from different individuals. The tendency of certain people to leave more (good shedder) or less (poor shedder) DNA in skin contact traces has already been observed many times. In addition to skin characteristics [4–7], a person’s age as well as the activity before contact, such as washing hands, can cause varying results regarding one and the same person [4, 5, 8–11].

**Table 1:** The amount of DNA in pg/µl obtained per swab, the median amount of DNA in pg/µl across all sampling sites per individual, and the amount of DNA in pg/µl per sampling site. 11 samples from 0 to 0.5 pg/µl; 20 samples between 0.5 and 1 pg/µl; 74 samples between 1 and 10 pg/µl; 31 samples between 10 and 100 pg/µl, 4 samples with more than 100 pg/µl

Subject	Amount of DNA in pg/μl right palm	Amount of DNA in pg/μl left palm	Amount of DNA in pg/μl back of hand	Amount of DNA in pg/μl upper arm	Amount of DNA in pg/μl neck	Median Amount of DNA in pg/μl across all sampling sites
2	0,5	2,2	3,6	0,5	0,8	0,8
4	0,3	0,3	0,6	1,9	6,4	0,6
5	0,5	0,5	0,6	2,4	6,4	0,6
7	13,5	7	1,8	0,8	17,6	7,0
8	82,1	123,8	10,4	3,3	570,6	82,1
9	1,6	5,3	3	3,3	20,1	3,3
10	0,4	1,4	0,8	0,4	10,2	0,8
11	16,3	5,8	7,4	4,2	2,8	5,8
14	2,4	0,6	1,2	0,9	3,5	1,2
15	9,3	6,6	26,5	12,1	3,7	9,3
17	0,2	0,2	0,5	0	14,2	0,2
18	0,3	1,2	0,2	1,4	2,1	1,2
19	7,6	7,7	15,1	89,2	21	15,1
23	1,8	3,4	3,2	0,9	1	1,8
25	35,4	45,6	3,4	1,9	3,6	3,6
27	8,4	20,6	25	18	58,5	20,6
28	3,6	6,2	0,6	0,7	22,6	3,6
33	2,7	1,3	2,6	0	4,2	2,6
34	9,1	1,4	4,9	1,6	4,9	4,9
35	1	0,9	0,5	0,3	6,9	0,9
36	17,7	29,3	8,3	30,5	464,3	29,3
39	11,6	2,8	2,5	3,4	7,4	3,4
40	1,3	1,7	1,3	0,6	9,4	1,3
42	4,1	5	1,3	1,1	9,2	4,1
45	1,3	3,8	0,7	3,8	106,7	3,8
48	1,4	2,9	5,3	1,7	84,8	2,9
50	0,5	4,4	12,5	1	37,5	4,4
53	19,5	40,2	17	3,8	19,7	19,5
Median DNA amountin pg/μl per sampling site	2,6	3,6	2,8	1,65	9,3	

The results also show that some persons shed widely varying amounts of DNA from different parts of the body. Swabs from the neck showed DNA amounts greater than 0.5 pg/µl for all patients and provided the greatest DNA yield in 19 of 28 tested individuals. Three of the remaining nine patients left the greatest DNA amount on swabs of each, the right or left hand, two on swabs of the back of their hand and one on the swab taken from the upper arm (see Table 1). Statistical comparison of the different swabbing sites regarding the DNA amounts obtained (Kruskal–Wallis test, significance level 5%) resulted in a *p*-value of 0.000 and thus in a significant difference. However, pairwise comparison of DNA amounts obtained from the skin swabs of the various sampling sites (Mann–Whitney *U* test, significance level 5%) showed no significant differences between samples taken from the right or left hand, the back of the hand, and the upper arm (data not shown). Only the swabs taken from the neck showed a significant difference compared to the samples from the right palm (*p*-value = 0.004), left palm (*p*-value = 0.007), back of the hand (*p*-value = 0.001), and upper arm (*p*-value = 0.000). Thus, as already described by Kamphausen et al. [12], for most people, the neck area provides more DNA material than other body regions.

#### STR typing

A total of 38 of the remaining 129 skin swabs examined were assigned to category A. All samples of person four were among those 38 samples. In ten of the remaining 26 subjects, all skin swabs produced could be classified in category B. In the remaining 16 patients, usable results were obtained for only 1 to 4 swabs.

In 76 of the total of 91 (83%) samples assigned to category B, alleles of other persons could be detected in addition to the recipient and donor alleles. The affected samples showed between two and 31 additional alleles in the 16 autosomal STR systems examined. Most frequently, one to ten additional signals were found (data not shown). No extraneous material was detectable on the remaining 15 swabs. All four swabs with DNA amounts greater than 100 pg/µl were among those samples. The remaining samples in this group showed a DNA amount of 1–10 pg/µl (four samples) and 10–100 pg/µl (seven samples), respectively. A correlation of the DNA amounts in all samples and the results regarding the number of additionally obtained alleles shows a Spearman-Rho correlation coefficient of − 0.202 with a *p*-value of 0.055 (significance level 5%) and consequently gives a very low negative, but non-significant correlation. This, together with the fact that no additional features were found on the four swabs with a DNA amount greater than 100 pg/μl, provides at least an indication that less foreign material can be detected on swabs with a higher DNA yield. It is well known that as the amount of DNA decreases, the number of drop-in events increases, which could explain at least part of these results. Moreover, this fact was also previously described by Goray et al. [13], where good shedders turned out to leave more of their own DNA on objects than foreign alleles and, in turn, poor shedders release less of their own DNA to their environment than foreign DNA. According to Goray et al. [13], this could possibly be due to the fact that poor shedders take up similar amounts of foreign DNA as good shedders but do not overlay it with their own DNA. Since STR typing only reliably detects mixtures up to a ratio of 1:20, foreign material is likely to be present on swabs with a high DNA yield, but it is not detectable in STR typing due to unbalanced mixture ratios.

All 91 samples assigned to category B showed both recipient and donor alleles in different proportions or mixture ratios. For nine of the skin swabs, mixtures could be obtained in which all recipient alleles were detectable. The donor’s alleles were detected almost completely or only partially (one to five missing alleles). On the other hand, in 22 samples, donor’s alleles were completely present in the mixtures and those of the recipient only incompletely (one to ten missing alleles). Donor’s as well as recipient’s complete DNA profiles were found in the mixtures of 34 samples in total. In the remaining 26 samples, neither the recipient nor the donor alleles could be completely detected.

Twenty-one skin swabs with complete profiles of the recipient and/or donor (three samples with the complete recipient’s profile, 14 with the complete donor’s profile, and four samples which showed complete profiles of both persons) showed admixtures caused by additional persons, observable either due to too many alleles per locus or unusually high signal intensities. These samples as well as the 26 samples in which neither the recipient’s nor the donor’s alleles could be completely detected were not further evaluated. The remaining 44 skin swabs were assigned to category C and the ratio of recipient to donor alleles was determined.

#### Determination of ratio of recipient’s to donor’s alleles

The 44 samples assigned to category C consisted of eight swabs from the right palm, ten from the left palm, seven from the back of the hand, four swabs from the upper arm, and 15 samples from the neck from a total of 18 different patients. Sixteen of these samples contained a DNA amount of 1–10 pg/µl, 24 between 10 and 100 pg/µl, and four of the swabs more than 100 pg/µl. The proportion of recipient’s to donor’s alleles of these samples was determined considering the peak areas. This showed a range of variation from 12–81% for recipient alleles and consequently from 19–88% for donor alleles (see Table 2). These results show that cells with donor alleles can be found on the prepared skin swabs. However, the preliminary test for blood was negative for all samples, suggesting that the samples contain other cells formed by the new bone marrow of the donor.

**Table 2:** The graphic shows 44 samples assigned to category C with the respective DNA quantity as well as the percentage of recipient and donor alleles

Subject	Sampling site	DNA amount in pg/µl	Percentage of recipient alleles in %	Percentage of donor alleles in %
27	Neck	58.5	12	88
53	Back of hand	17	13	87
50	Back of hand	12.5	15	85
40	Neck	9.4	15	85
27	Back of hand	25	17	83
8	Left palm	123.8	18	82
27	Upper arm	18	20	80
8	Neck	570.6	24	76
45	Neck	106.7	25	75
19	Upper arm	89.2	26	74
27	Left palm	20.6	27	73
7	Neck	17.6	28	72
27	Right palm	8.4	28	72
48	Neck	84.8	32	68
53	Right palm	19.5	32	68
34	Back of hand	4.9	33	67
25	Neck	3.6	40	60
33	Back of hand	2.6	41	59
8	Upper arm	3.3	42	58
8	Back of hand	10.4	45	55
34	Right palm	9.1	47	53
25	Left palm	45.6	48	52
35	Neck	6.9	48	52
36	Neck	464.3	48	52
11	Left palm	5.8	49	51
9	Left palm	5.3	51	49
36	Right palm	17.7	51	49
7	Right palm	13.5	52	48
36	Upper arm	30.5	54	46
53	Left palm	40.2	55	45
7	Left palm	7	55	45
9	Neck	20.1	56	44
8	Right palm	82.1	57	43
42	Neck	9.2	58	42
19	Neck	21	59	41
25	Right palm	35.4	59	41
34	Neck	4.9	59	41
50	Neck	37.5	61	39
17	Neck	14.2	62	38
19	Back of hand	15.1	64	36
42	Left palm	5	67	33
36	Left palm	29.3	74	26
19	Right palm	7.6	81	19
19	Left palm	7.7	81	19

Since skin swabs were collected from the epidermis’ surface, which consists mainly of keratinocytes in this area, this result is somewhat surprising. Only the so-called Langerhans cells (LC) located in the lower stratum spinosum and first described by Paul Langerhans in 1868 should be mentioned as cells of the epidermis with a myeloid background while dermal dendritic cells are located beyond the basal membrane, in the dermis [14, 15]. While the function of LC is still being discussed, their myeloid origin is proven in mice [16–18] as well as in humans [19, 20]. As antigen-representing cells of the epidermis, they react to a stimulus by differentiating and migrating to the local lymph nodes [21] and play a role in coordinating an immune response [16, 22, 23]. Remarkably, they appear to renew themselves locally via mitosis rather than upon migration of hematopoietic cells under normal conditions [24]. Under inflammatory conditions, on the other hand, the neonatal, long-lived LC are supplemented or replaced by fresh, short-lived LC, which mature from the pool of monocytes [24–28]. Since those monocytes develop from the bone marrow, they exhibit donor alleles after alloHSCT. The extent to which either this or the different subclasses are clinically relevant, e.g., with regard to the occurrence of graft versus host disease, has not been finally clarified. Graft versus host disease (GvHD) is a crucial factor for mortality and morbidity after alloHSCT and was first described in mice by Barnes and Loutit [29] and defined by Billingham [30]. Acute GvHD (aGvHD) primarily infests the skin but also the colon and liver [31]. In contrast, chronic GvHD (cGvHD), which occurs later, is a syndrome that shows significantly greater variability regarding the organs involved [32].

The wide range of variation between 19–88% of donor alleles and 81–12% of recipient alleles obtained in samples collected at least 180 days after alloHSCT and examined here shows that different amounts of recipient’s cells and cells with donor’s alleles were found on the prepared swabs. Assuming that after this time all LC have been replaced by donor cells [33, 34], the recipient portion would consist of an admixture of DNA from non-myeloid cells such as keratinocytes. Consequently, this would be an indication that not all individuals shed an equal number of skin cells or immune cells located in the skin upon contact.

However, the wide variation can also be explained by an incomplete chimerism of these cells. Divergent observations regarding a chimerism of the skin were made based on different models. Thus, complete donor chimerism of the LC was described after 84–100 days after dose-reduced conditioning, whereas a study by Perreault et al. [35] showed that LC of the host could be detected up to a year after transplantation. The time span in which this exchange takes place also seems to depend on the amount of transplanted T-cells from the donor. Merad et al. [36] showed that in mice transplanted with allogeneic bone marrow or with purified hematopoietic stem cells, Langerhans cells of the donor could not be detected in the skin even 18 months after transplantation. The exchange of these immune cells only took place after donor T cells were added to the transplanted bone marrow. Thus, both the post transplantation time span and the proportion of T-cells in the transplant may be responsible for the variability of donor allele to recipient allele ratios among the tested patients. Potential GvHD may also have an impact. The association of antigen-presenting cells [37] or specific LC [35] with GvHD was postulated early on, but is not yet well understood. Whereas Merad et al. [36] state that skin GvHD can be prevented by LC depletion, other results suggest that GvHD does not require classic LC [38], aGvHD is triggered by donor monocytes [39], and that skin chimerism can occur regardless of cGvHD [40]. The impact of different conditioning regimes or the immunosuppressive treatment prior to transplantation on skin chimerism is yet another issue demanding further clarification.

When comparing the DNA quantities of all 44 samples assigned to category C with the respective proportions of recipient and donor alleles, a Spearman-Rho correlation coefficient of –0.212 (recipient alleles) and 0.212 (donor alleles) was obtained with a *p*-value of 0.167 (significance level 5%), thus yielding only a very slight negative (DNA quantity/proportion of recipient alleles) and positive (DNA quantity/proportion of donor alleles), non-significant correlation. Consequently, the recipient and donor proportion has no significant effect on the DNA quantity. There is only a slight tendency suggesting that a higher DNA amount could be yielded when the proportion of donor cells is larger.

**Fig. 1:**
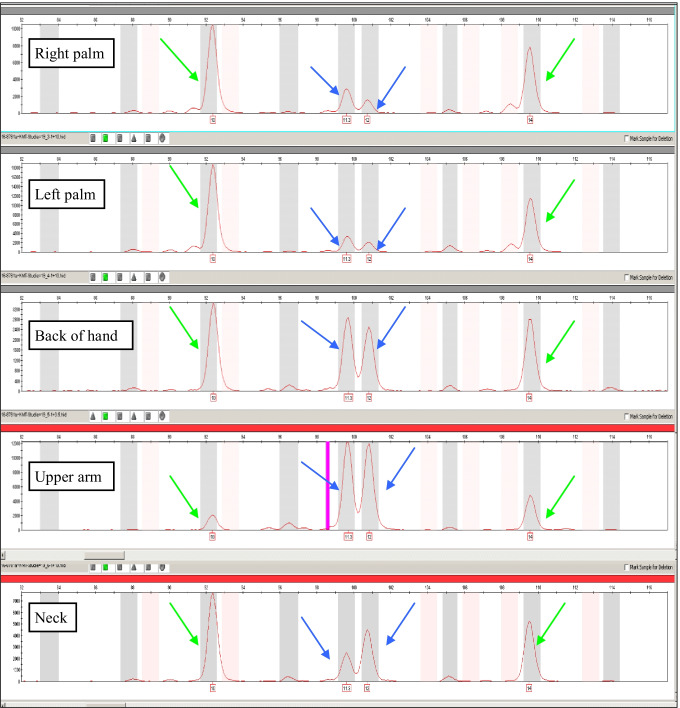
The figure shows a closeup on marker SE33 of electropherograms resulting from swabs of the right palm, left palm, back of the hand, upper arm and neck of patient 19. The yellow green arrows are the recipient alleles and the blue arrows are the donor alleles

In only three cases (patients 8, 19, and 27), all skin swabs could be assigned to category C and the calculation of recipient to donor allele ratio could subsequently be carried out (see Table 3). Patient 27 showed allele mixtures with identically dominant components in all samples that could be assigned to the donor. The proportion of recipient alleles in these samples was only 12–28%. With 18–57% of recipient alleles in patient 8, typing showed either balanced allele mixtures of recipient and donor alleles or mixtures with a main component of donor alleles. Of the three subjects, person 19 showed the largest variation of recipient alleles from 26–81% and thus of donor alleles from 19–74%. In swabs of the right and left palms, 81% of recipient alleles could still be detected, which was reflected in derivable dominant alleles in the respective mixture. Typing the swabs of the neck and the back of the hand resulted in balanced allele mixtures with a percentage of 59% and 64% of recipient alleles, respectively. Finally, a dominant proportion of donor alleles were found in the swab of the upper arm with a minor component of 26% of recipient alleles (see Fig. 1). Thus, the distribution between recipient cells and cells with donor DNA not only differs between individual patients, but also within one and the same person. Depending on the body region, large variations between the proportions of recipient and donor alleles could occur. This could also be related, as already mentioned above, to the fact that different compositions of delivered skin cells and cells with donor alleles are found in swabs of the different collection sites.

**Table 3:** Percentage of recipient and donor alleles for the five body swabs of patients 8, 19, and 27

Subject	Sample	Percentage of recipient alleles in %	Percentage of donor alleles in %
8	Right palm	57	43
	Left palm	18	82
	Back of the hand	45	55
	Upper arm	42	58
	Neck	24	76
19	Right palm	81	19
	Left palm	81	19
	Back of the hand	64	36
	Upper arm	26	74
	Neck	59	41
27	Right palm	28	72
	Left palm	27	73
	Back of the hand	17	83
	Upper arm	20	80
	Neck	12	88

However, the samples collected could also vary regarding proportions of donor and recipient LC. This may be due to a variable kinetics with which recipient cells are replaced by donor cells. This means that the exchange does not take place evenly but at a different pace or at different times in different skin parts. The type of transplantation may also play a role here. According to Merad et al. [36], transplantations without donor T cells might initially not lead to an exchange of immune cells. The recipient’s LC would accordingly remain in the skin until an exchange of cells is initiated by inflammatory reactions. Because an inflammatory stimulus mostly occurs locally, differences in the distribution of immune cells of the recipient and donor would result in the different skin areas. A local or generalized GvHD or different conditioning regimes may well play a role here, as well.

## Conclusion and outlook

This study shows that examined individuals who received an alloHSCT showed both recipient and donor alleles in all skin swabs suitable for STR typing. These results prove that not only skin cells like keratinocytes but also cell material freshly produced from the donor’s bone marrow can be found on the swabs taken from skin. Furthermore, the percentage of donor alleles of up to 88% shows that at least in a part of the produced skin swabs, these myeloid cells represent the majority of the DNA-providing cell material.

The application of alloHSCT increases. Combined with an increased survival rate, this results in a constantly growing number of transplanted and thus chimeric patients. However, this group of individuals still contributes to a population at a small scale. Consequently, involvement of such a person in a forensically relevant case scenario will remain a rather special case in routine forensic practice. Nevertheless, the results of this study show that an alloHSCT also plays a role in touch DNA and that the question of which cell material is left behind in these traces has not yet been fully clarified.

The use of specific antibodies could provide information regarding the extent to which LC are found on skin swabs and whether there are differences between individual persons or body regions, particularly with regard to the number of cells. In case of a positive test result, one could try to separate the immune cells of persons who have received an alloHSCT in order to specifically create DNA profiles from these cells. This could provide information about whether all recipient LC are being replaced by donor cells or whether there are mixtures here as well.

Both invasiveness (biopsies) and the complexity of the examination have so far prevented a broad data collection in clinical studies regarding skin chimerism of the LC after an alloHSCT. STR typing of easy to collect, non-invasive skin swabs could provide the basis for a broader data collection at least for those who shed sufficient amounts of cell material. For example, one could investigate to what extent the exchange of recipient LC with donor LC correlates with the post transplantation time span and whether other factors, such as the presence or absence of donor T-cells, play a role. The method applied in this study could also help investigate a possible correlation of the exchange of LC with the occurring of GvHD. A better understanding of dermal processes leading to GvHD is of great clinical interest. Insights as to whether the exchange of recipient LC with donor LC can be interpreted as a harbinger or as a result of an inflammation could contribute to a prognosis regarding both occurrence and course of an acute or chronic GvHD of the skin as well as to monitor the response to therapy.
